# ^1^H, ^13^C and ^15^N assignment of self-complemented MrkA protein antigen from *Klebsiella pneumoniae*

**DOI:** 10.1007/s12104-024-10185-3

**Published:** 2024-07-17

**Authors:** Valentina Monaci, Gianmarco Gasperini, Lucia Banci, Francesca Micoli, Francesca Cantini

**Affiliations:** 1 Magnetic Resonance Center – CERM, University of Florence, Via Luigi Sacconi 6, Sesto Fiorentino, 50019 Florence, Italy; 2 Department of Chemistry, University of Florence, Via della Lastruccia 3, Sesto Fiorentino, 50019 Florence, Italy; 3 GSK Vaccines Institute for Global Health (GVGH), Via Fiorentina 1, 53100 Siena, Italy; 4 GSK, Via Fiorentina 1, 53100 Siena, Italy

**Keywords:** NMR resonance assignment, *mrkA*, *Klebsiella pneumoniae*, Self-complemented monomer, Type 3 fimbriae, Heteronucelar relaxation data, Protein antigen

## Abstract

Klebsiella pneumoniae (Kp) poses an escalating threat to public health, particularly given its association with nosocomial infections and its emergence as a leading cause of neonatal sepsis, particularly in low- and middle-income countries (LMICs). Host cell adherence and biofilm formation of Kp is mediated by type 1 and type 3 fimbriae whose major fimbrial subunits are encoded by the *fimA* and *mrkA* genes, respectively. In this study, we focus on MrkA subunit, which is a 20 KDa protein whose 3D molecular structure remains elusive. We applied solution NMR to characterize a recombinant version of MrkA in which the donor strand segment situated at the protein’s N-terminus is relocated to the C-terminus, preceded by a hexaglycine linker. This construct yields a self-complemented variant of MrkA. Remarkably, the self-complemented MrkA monomer loses its capacity to interact with other monomers and to extend into fimbriae structures. Here, we report the nearly complete assignment of the ^13^C,^15^N labelled self-complemented MrkA monomer. Furthermore, an examination of its internal mobility unveiled that relaxation parameters are predominantly uniform across the polypeptide sequence, except for the glycine-rich region within loop 176–181. These data pave the way to a comprehensive structural elucidation of the MrkA monomer and to structurally map the molecular interaction regions between MrkA and antigen-induced antibodies.

## Biological context

Neonatal sepsis is a major cause of death across low- and middle-income countries (LMICs) (Milton et al. 2022). These infections, occurring in newborns, are acquired both in communities and in health-care facilities (Zaidi et al. 2005). *Klebsiella pneumoniae* (Kp) has been identified by different surveillance networks as a leading cause of neonatal sepsis (Sands et al. 2021). Kp is a gram-negative, encapsulated bacterium, belonging to the *Enterobacteriaceae* family, often found in a variety of environmental niches (Bagley 1985). Kp produces several biomolecules that are essential for virulence, including fimbriae that aid in the initial colonization of the host and capsular polysaccharides that protect the organism from phagocytosis, complement and inhibit macrophage differentiation (Alcántar-Curiel et al. 2013). Fimbriae are typically extracellular appendages with 0.5–10 μm length and 2–8 nm width, which are encoded by the *mrk* gene cluster (*mrk*ABCDF) that is comprised of five genes encoding the structural and assembly components of the appendages (Murphy and Clegg 2012). Two major adhesive fimbriae structures are responsible for adherence of Kp to eukaryotic epithelial cells: the mannose-sensitive type 1 fimbriae composed of a major fimbrial FimA subunit and a minor tip adhesin FimH; and the mannose-resistant type 3 fimbriae, composed of the major fimbrial subunit MrkA and the minor tip adhesin MrkD (Gerlach et al. 1988, 1989; Old et al. 1985). The type 3 fimbriae are believed to be assembled using the chaperone/usher pathway used by a variety of fimbrial systems. In fact, exploring other fimbrial gene clusters MrkB and MrkC are recognized to belong to the family of periplasmic chaperones and scaffolding proteins implicated in fimbrial assembly (Allen et al. 2012; Morrissey et al. 2012; Thanassi et al. 1998). In this assembly pathway, fimbrial subunits are transported via the general secretory pathway to the periplasm where a chaperone, in the case of type 3 fimbriae encoded by *mrk*B, forms a complex with the fimbrial subunit proteins. This complex is directed to the scaffolding protein MrkC, located at the outer membrane. Fimbrial assembly is accomplished by addition of MrkA subunits to the growing appendage and MrkD as its tip (Allen et al. 2012; Morrissey et al. 2012). Previous studies have indicated that MrkF may be randomly incorporated into the growing fimbrial appendage to confer stability or may serve as an adaptor protein for MrkD and MrkA; its precise location in the fimbriae is unknown (Huang et al. 2009; Murphy and Clegg 2012). MrkA, a 20 KDa protein with a high conserved amino acid sequence among the *Enterobacteriaceae* strains analyzed so far (Wang et al. 2017), has been recognized as the common protein antigen expressed by the majority of Kp strains with the function of biofilm formation and establishment of infection (Boddicker Jennifer et al. 2006; Langstraat et al. 2001; Schroll et al. 2010). To date, its 3D molecular structure is not known. Here we take the challenge to assign the NMR signals of this protein, as first step toward its more in-depth structural characterization. Such studies are essential to investigate this protein as a potential antigen and to look into its mechanism of action. We assign the recombinant form of the protein, by generating a self-complemented variant of MrkA, which is extended at the C-terminus by a hexaglycine linker followed by a second copy of the MrkA donor strand (residues 1–20 in wild-type (wt) MrkA). The donor strand is a key element for fimbrial proteins (Poole et al. 2007): it is reported that the elongation to a fimbriae is due to the interaction via donor strand complementation among the subunits, where the incomplete, immunoglobulin-like fold of each subunit is complemented by an N-terminal donor strand of the subsequent subunit (Walczak et al. 2014; Żyła et al. 2019).

## Methods and experiments

### Design, expression and purification of the self-complemented MrkA monomer

**Fig. 1:**
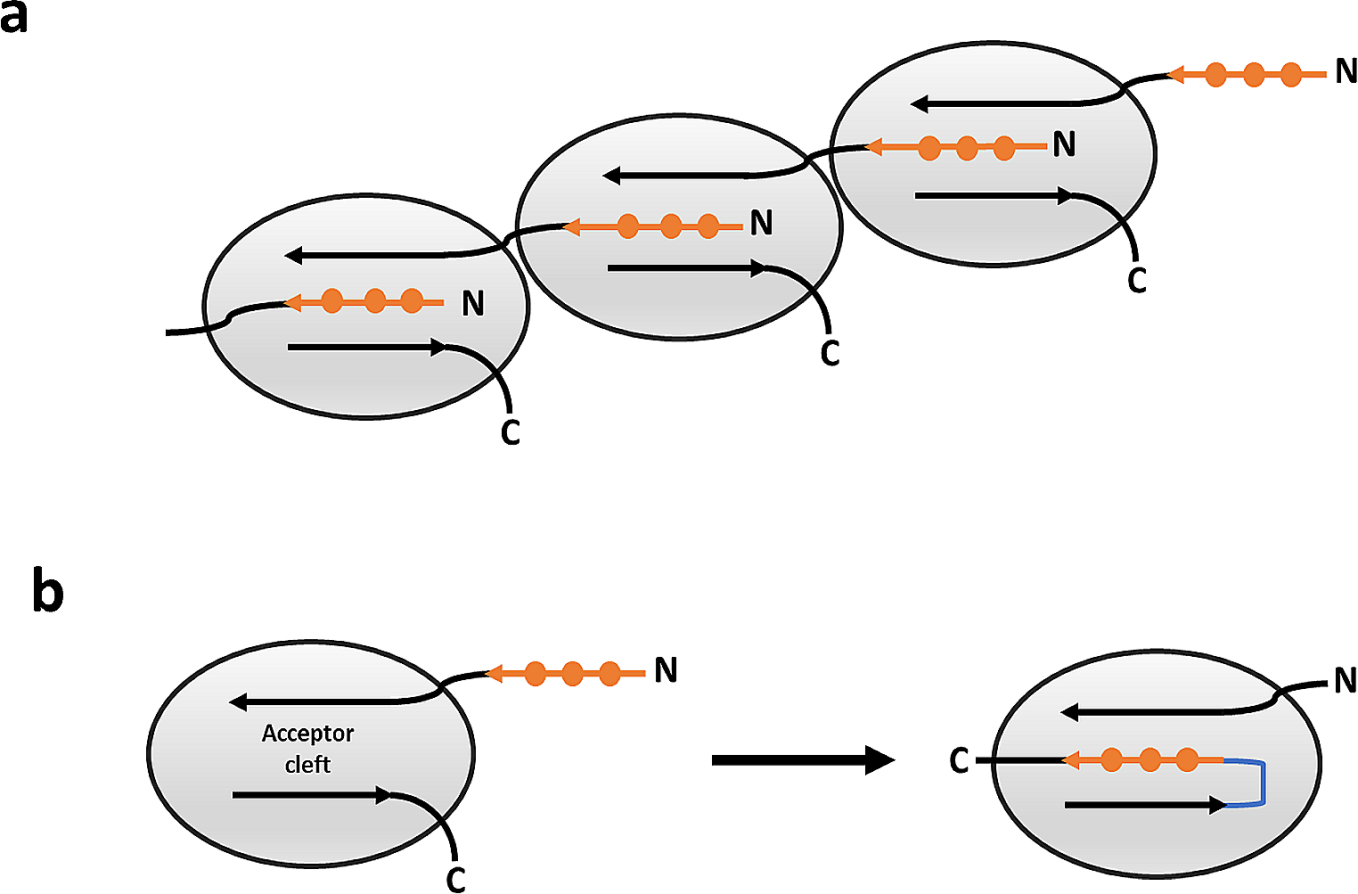
**(a)** Natural assembly of fimbrial monomers: the N-terminus donor strand (in orange) of a monomer is inserted in the acceptor cleft of the following monomer, forming a pearl necklace structure. **(b)** Schematic representation of the self-complemented MrkA monomer, in which the donor strand (in orange) is moved from the N-terminus to the C-terminus, preceded by a glycine stretch (in blue) inserted between the normal C-terminus and the complementing strand moved to the C-terminus. In our protein construct, the numbering of the protein is such that Ser 42, Gln 202 and the N-terminus stretch, Ala 23-Ser 42, of the WT protein (P12267 · FM3_KLEPN), matches with Ser 15, Gln 175 and the C-terminus stretch, Ala 182-Ser 201, in our sequence construct.

The donor strand displacement strategy is applied to MrkA of Kp in order to obtain a self-complemented monomer not able to elongate to a fimbria Fig. 1. Specifically, the donor strand (first 20 aa in the mature protein after leader sequence cleavage) present at the N-terminus is moved to the C-terminus and a hexaglycine linker is added between the normal C-terminus and the complementing strand moved to the C-terminus to let the donor strand to assume an antiparallel orientation within the beta sheet as it has been already observed for inter-molecular donor strand complementation in FimA polymers (Żyła et al. 2019).

**Fig. 2:**
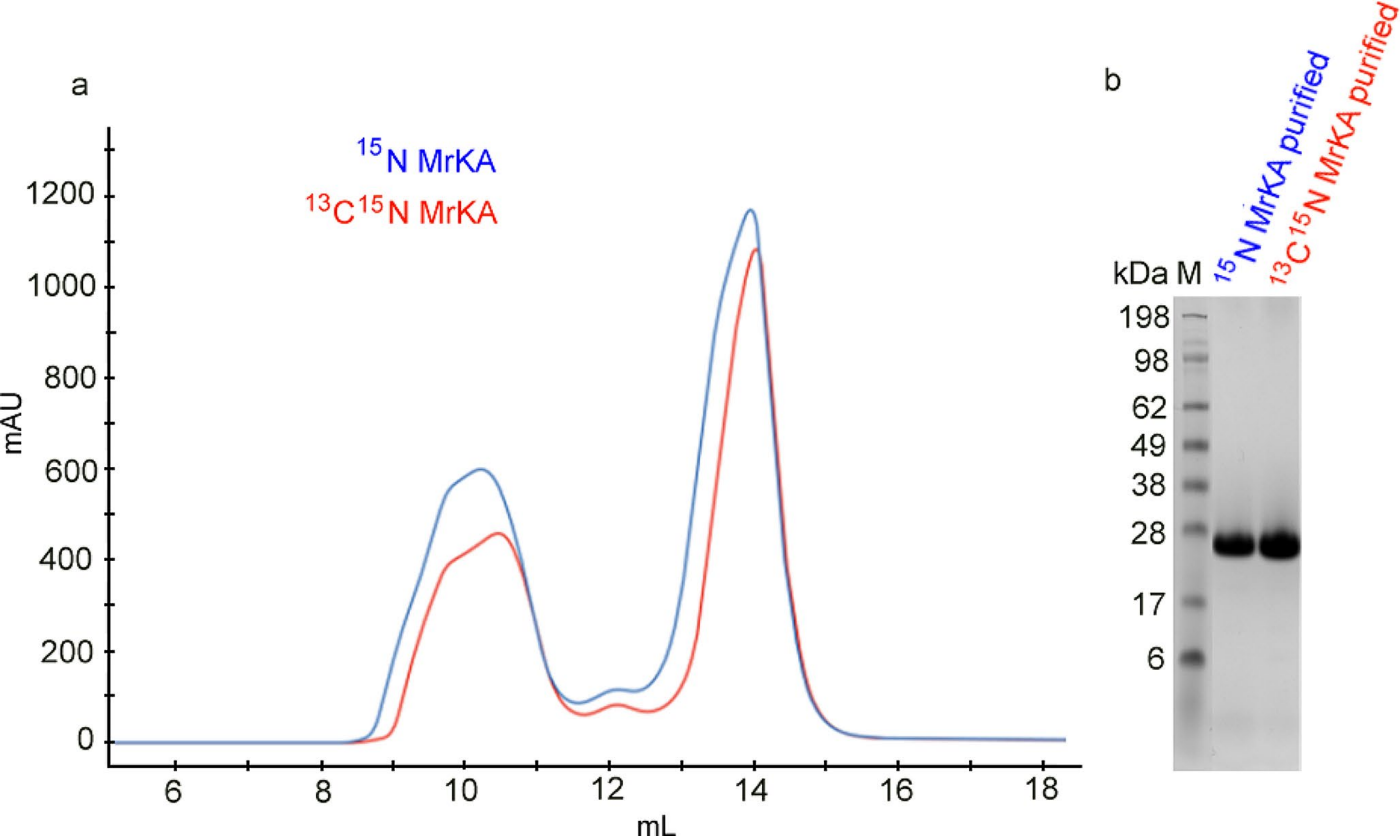
SEC profiles (**a**): elution with PBS. Separation of labelled MrkA monomer from aggregates (elution at 10 mL). Collection of the labelled MrkA monomer (elution at around 14 mL) is confirmed by SDS-PAGE analysis (**b**) of the purified ^15^N- and ^13^C-^15^ N MrkA samples

The corresponding gene of self-complemented MrkA monomer (preceded by a methionine and a 10-histidine tag) is inserted into a pET29b (+) Twist Bioscience plasmid, resulting in a construct of 201 residues. The plasmid is used to transform *E. coli* BL21 (DE3) competent cells by ThermoFisher Scientific. Cell growth is performed in ^15^N and ^13^C-^15^N ISOGRO medium by Sigma-Aldrich (5 g/L; addition of 100 g/L K_2_HPO_4_, 50 g/L KH_2_PO_4_, 50 g/L MgSO_4_ and 37 g/L CaCl_2_) at 30 °C in order to obtain both mono-labeled and double-labeled MrkA monomer. When the culture reaches an OD_600_ of 0.8–1, 1 mM IPTG is added to induce protein expression, and the cells are incubated at 20 °C overnight. Cells are harvested and lysed using CelLytic Reagent by Sigma-Aldrich, following the manufacturer’s instructions. After incubation, the lysate is centrifuged and the supernatant containing the soluble protein fraction is diluted with 50 mM sodium phosphate, 500 mM NaCl, 30 mM imidazole pH 7.4, filtered using a 0.22 μm filter and then loaded in a HisTrap FF affinity chromatography column by Cytiva. The column is then washed with an imidazole gradient and MrkA protein eluted with 500 mM imidazole, pH 7.4. A size exclusion chromatography step is finally performed to ensure the removal of aggregates from the final protein sample. A Superdex 75 Increase prepacked column by Cytiva has been chosen with an isocratic elution in 50 mM sodium phosphate, 100 mM NaCl pH 7.0. Peak fractions are pooled together and checked by SDS-PAGE gel analysis to confirm the monomeric form of the proteins and their purity (Fig. 2).

### NMR spectroscopy

All NMR experiments used for resonances assignment of MrkA are recorded on a Bruker AVANCE 950 MHz spectrometer on ^13^C-^15^ N-labeled sample. Heteronuclear relaxation measurements, ^15^N- R_1_, ^15^N-R_2_ and ^1^H-^15^ N NOE are recorded on a Bruker AVANCE 500 MHz spectrometer equipped with a triple resonance cryoprobe TXI on a ^15^N-MrkA sample. For ^1^H-^15^ N NOE measurements, delays of 5s are used between repetitions of the pulse sequence. For ^15^N- R_1_ and ^15^N -R_2_ 3s of delay is used. Amide resonances are integrated using CARA software (Keller et al. 2006) and ^15^N- R_1_ and ^15^N-R_2_ values are obtained by fitting peak intensities using single exponential decay:

$${I}_{(t)} = {{I}_{0}} {{exp} {(-{t}/{T}_{1,2})}}$$

where I_(t)_ is the peak intensity, t is the time, and I_0_ is the intensity at time 0 using ORIGIN software (Origin (Pro), Version 2023 OriginLab Corporation, Northampton, MA, USA). The analysis of the uncertainties of the ^15^N- R_1_ and ^15^N-R_2_ values is carried out by comparing the peak heights on duplicate spectra at 10 ms (shortest value of relaxation delay). The heteronuclear steady-state and ^1^H-^15^ N NOE values are obtained from the ratios of peak intensities in the saturated spectrum to those in the unsaturated spectrum. The radio frequency pulses, carrier frequencies, acquisition and processing parameters of all the NMR experiments needed for the backbone and side-chain resonances assignment are reported in Table 1.

The NMR samples has a protein concentration of about 400 µM for ^13^C-^15^ N-MrkA and 450 µM for ^15^N-MrkA in 50 mM sodium phosphate, 100 mM NaCl pH 7.0 and 10% (v/v) D_2_O. All NMR spectra for resonances assignment are collected at 298 K, processed using the standard Bruker software Topspin (version 4.3) and analyzed through the CARA program (Keller et al. 2006).

### Extent of assignments and data deposition

**Fig. 3:**
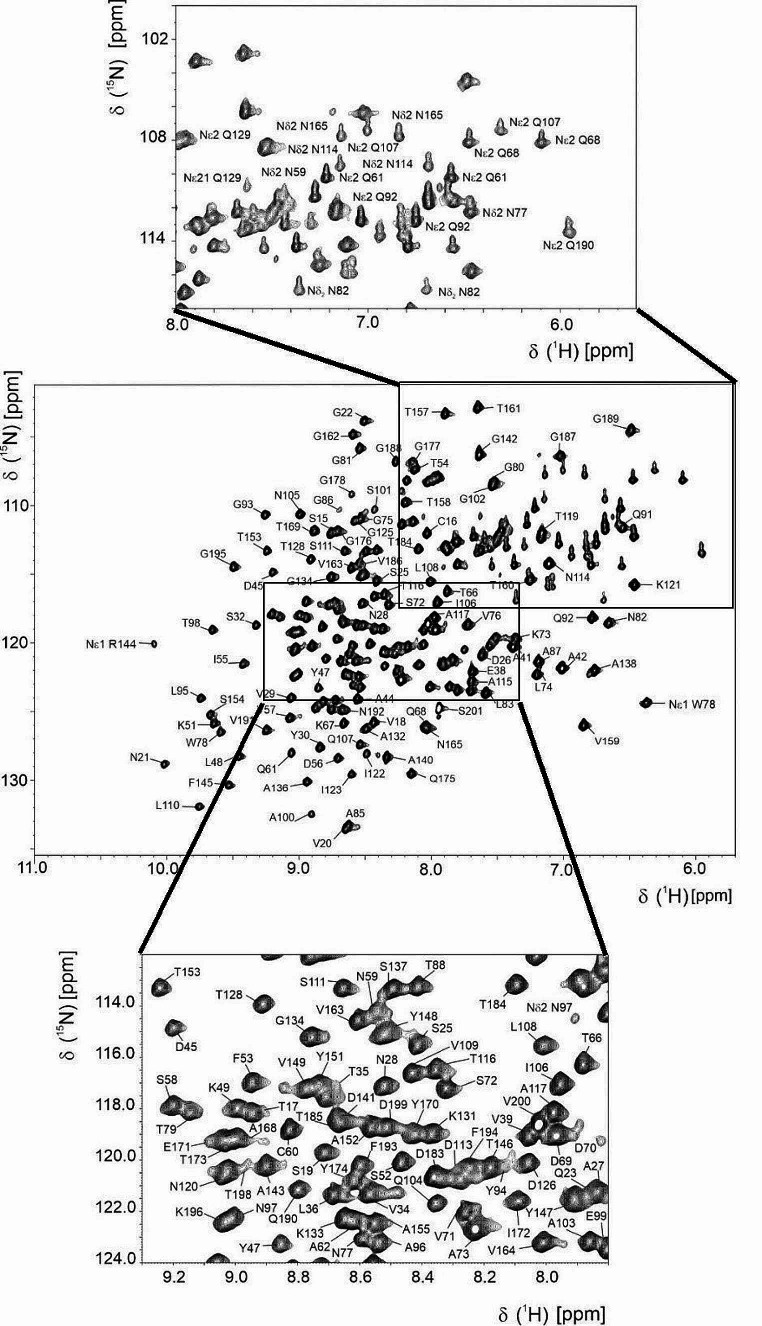
2D ^15^N HSQC showing the complete backbone NH assignments of MrkA at 298 K. For sequence numbering see legend of Fig. 1

The ^1^H- ^15^ N HSQC spectra of MrkA show well-dispersed resonances indicative of an essentially folded protein (Fig. 3). The backbone resonance assignment is obtained from the analysis of the triple resonance spectra. 181 out of the expected 196 ^15^N backbone amide resonances are assigned. The amide resonances are missing for residues Met 1, Gly 2, Ser 3, His 4- His 13, Gly 179 and Gly 180. The assignment of the aliphatic side chain resonances is performed through the analysis of 3D CC(CO)NH and (H)CCH-TOCSY spectra, together with ^15^N-NOESY-HSQC and ^13^C-NOESY-HSQC spectra. The assignment of the aromatic spin systems is performed with 2D NOESY and TOCSY maps and a 3D ^13^C-NOESY-HSQC spectrum with the carrier centered in the aromatic region at 130 ppm. In total, the resonances of 81% of carbon atoms, 92% of backbone nitrogen atoms, and 92% of protons are assigned, leaving only Met 1, Gly 2, Ser 3, His 4–13 and Gly 179 completely unassigned.

**Fig. 4:**
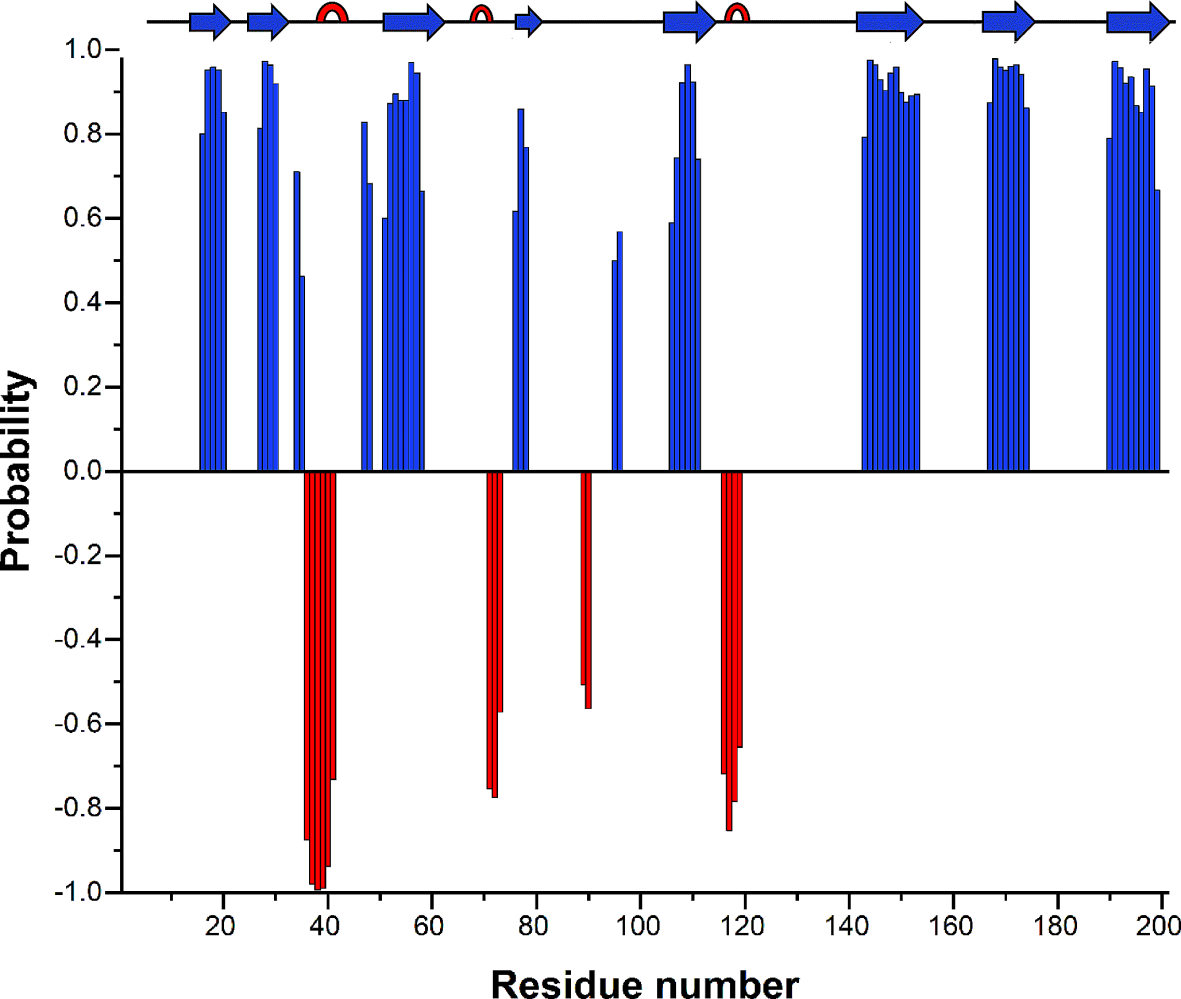
Chemical shift-based prediction of the secondary structure elements by TALOS-N. Blue bars represent β-strands and red bars α-helices. For sequence numbering see legend of Fig. 1

We determine the amino acid specific secondary structure properties of MrkA from the assigned backbone chemical shifts (HN, Cα, Cβ, CO, N); using TALOS-N program (Shen and Bax 2013) we reveal that the secondary structure of MrkA comprises three small α-helices and eight β-strands (Fig. 4).

### ^15^N-relaxation experiments

**Fig. 5:**
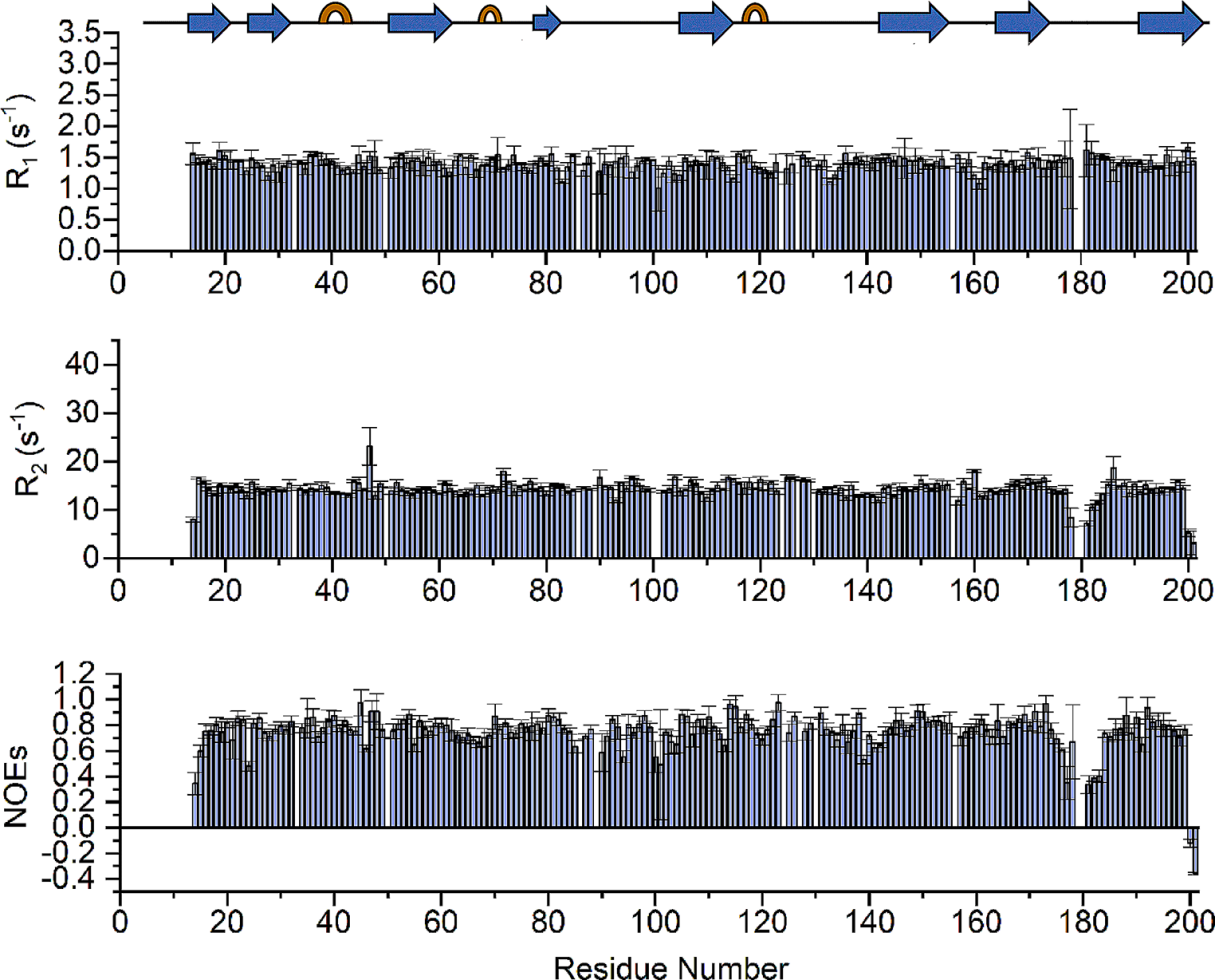
R_1_, R_2_, and ^1^H-^15^ N NOE values versus the residue number as obtained from the ^15^N relaxation data measured at 500 MHz

Reliable ^15^N R_1_, R_2_, and ^1^H-^15^ N NOE values, which provide information on internal mobility, are obtained for 181 of the 196 assigned backbone NH resonances. Peaks are integrated using CARA software and the relaxation rates are calculated using EXCEL/ORIGIN software. R_1_, R_2_, and ^1^H-^15^ N NOE average values of MrkA are 1.41 ± 0.1 s^− 1^, 14.5 ± 0.73 s^− 1^, and 0.77 ± 0.06, respectively. The relaxation parameters are essentially homogeneous along the entire polypeptide sequence with exception of glycine stretch located in loop 176–180 (Fig. 5). This is not surprising as it is the linker added to allow the donor strand to assume the correct orientation within the beta sheet. The correlation time for molecule reorientation (τ_m_), estimated from the R_2_/R_1_ ratio, is 10.2 ± 0.7 ns, as expected for a protein of this size in a monomeric state.

## Conclusion

The complete assignment of the bacterial protein antigen is a key step in the full characterization of MrkA. Thanks to this preliminary work performed with solution NMR spectroscopy, we set the basis for solving the NMR solution structure of this antigen. In perspective, structural studies are essential to characterize and better design the protein as antigen in vaccinology.

**Table 1:** Acquisition parameters for NMR experiments performed on MrkA

Experiments	Time domain data size			Spectral width			T (K)	ns	D1	Acquisition time			Magnetic Field
	(points)			(ppm)					(s)	(ms)			(MHz)
	t_1_	t_2_	t_3_	F1	F2	F3				F1	F2	F3	
**HNCA**	96	48	2048	30 (^1^H)	38 (^15^N)	14 (^13^C)	298	8	0.25	4.8	6.1	92	950
**HNCO**	88	48	2048	16 (^13^C)	38 (^15^N)	14 (^1^H)	298	4	0.25	9.9	6.3	92	950
**HNCOCA**	96	48	2048	30 (^1^H)	38 (^15^N)	14 (^13^C)	298	16	0.25	4.8	6	92	950
**HNCACO**	88	48	2048	16 (^1^H)	38 (^15^N)	14 (^13^C)	298	24	0.25	9	6	90	950
**15 N-separated NOESY**	256	68	2048	14 (^1^H)	32 (^13^C)	14 (^1^H)	298	8	1	7.6	1.4	61	950
**13 C-separated NOESY**	256	68	2048	14 (^1^H)	32 (^13^C)	14 (^1^H)	298	4	1	7.6	1.4	61	950
**HNCACB**	96	48	2048	80 (^13^C)	38 (^15^N)	14 (^1^H)	298	24	1	2.5	6.2	77	950
**CBCACONH**	96	48	2048	80 (^13^C)	38 (^15^N)	14 (^1^H)	298	16	1	2.5	6.2	77	950
**HCCH TOCSY**	1	64	2048	80 (^13^C)	80 (^13^C)	14 (^1^H)	298	16	1.2	7.6	1.4	61	950
^**15**^ **N-HSQC**	1024	128	-	16 (^1^H)	38 (^15^N)	-	298	8	1.2	64	16	-	500–950
^**15**^ **N -R**_**1**_^**15**^ N-HSQC*	1024	128	-	16 (^1^H)	38 (^15^N)	-	298	16				-	500
^**15**^ ** N-R**_**2**_ -HSQC*	1024	128	-	16 (^1^H)	38 (^15^N)	-	298	16				-	500

* A series of twelve ^15^N -R_1_^15^N-HSQC experiments are recorded using period of 0.010 s, 0.04 s, 0.08 s, 0.125 s, 0.200 s, 0.370 s, 0.500 s, 0.675 s, 0.800 s, 1 s, 1.55 s and 2.5 s. A series of eleven ^15^N-R_2_-HSQC experiments are recorded using period of 8.48 ms, 16.96 ms, 33.92 ms, 50.88 ms, 67.84 ms, 101.76 ms, 135.64 ms, 152.64 ms, 186.56 ms, 203.52 ms and 220.48 ms

## Data Availability

The chemical shift values for ^1^ H ^15^ N and ^13^ C resonances of MrkA are deposited BioMagResBank BMRB, under access number 52205.
